# Use of Multivariable Mendelian Randomization to Address Biases Due to Competing Risk Before Recruitment

**DOI:** 10.3389/fgene.2020.610852

**Published:** 2021-01-15

**Authors:** C. M. Schooling, P. M. Lopez, Z. Yang, J. V. Zhao, Shiu Lun Au Yeung, Jian V. Huang

**Affiliations:** ^1^Graduate School of Public Health and Health Policy, City University of New York, New York, NY, United States; ^2^School of Public Health, Li Ka Shing Faculty of Medicine, The University of Hong Kong, Hong Kong, China; ^3^Department of Epidemiology and Biostatistics, School of Public Health, Faculty of Medicine, Imperial College London, London, United Kingdom; ^4^Singapore Institute for Clinical Sciences (SICS), The Agency for Science, Technology and Research (A^∗^STAR), Singapore, Singapore

**Keywords:** selection bias, competing risk, Mendelian randomization, shared etiology, instrumental variable analysis

## Abstract

**Background:** Mendelian randomization (MR) provides unconfounded estimates. MR is open to selection bias when the underlying sample is selected on surviving to recruitment on the genetically instrumented exposure and competing risk of the outcome. Few methods to address this bias exist.

**Methods:** We show that this selection bias can sometimes be addressed by adjusting for common causes of survival and outcome. We use multivariable MR to obtain a corrected MR estimate for statins on stroke. Statins affect survival, and stroke typically occurs later in life than ischemic heart disease (IHD), making estimates for stroke open to bias from competing risk.

**Results:** In univariable MR in the UK Biobank, genetically instrumented statins did not protect against stroke [odds ratio (OR) 1.33, 95% confidence interval (CI) 0.80–2.20] but did in multivariable MR (OR 0.81, 95% CI 0.68–0.98) adjusted for major causes of survival and stroke [blood pressure, body mass index (BMI), and smoking initiation] with a multivariable Q-statistic indicating absence of selection bias. However, the MR estimate for statins on stroke using MEGASTROKE remained positive and the Q statistic indicated pleiotropy.

**Conclusion:** MR studies of harmful exposures on late-onset diseases with shared etiology need to be conceptualized within a mechanistic understanding so as to identify any potential bias due to survival to recruitment on both genetically instrumented exposure and competing risk of the outcome, which may then be investigated using multivariable MR or estimated analytically and results interpreted accordingly.

## Introduction

Mendelian randomization (MR), i.e., instrumental variable analysis with genetic instruments, is an increasingly popular and influential analytic technique (Davies et al., 2018; Taubes, 2018), which can be used to investigate causal effects even when no study including both exposure and outcome of interest exists. Invaluably, MR studies have provided estimates more consistent with results from randomized controlled trials (RCTs) than conventional observational studies, even foreshadowing the results of major trials (Holmes et al., 2017). MR studies are often presented as observational studies analogous to RCTs (Davey Smith and Ebrahim, 2005; Burgess et al., 2012) because they take advantage of the random assortment of genetic material at conception, while observational studies are open to biases from confounding and selection bias (Bareinboim and Pearl, 2016). Instrumental variable analysis is described in health research as addressing confounding (Greenland, 2000; Maciejewski and Brookhart, 2019), i.e., bias from common causes of exposure and outcome (Bareinboim and Pearl, 2016). MR is currently described as “less likely to be affected by confounding or reverse causation than conventional observational studies” (Davies et al., 2018).

Mendelian randomization was originally thought to be less open to selection bias than conventional observation studies (Smith and Ebrahim, 2004). Selection bias is now increasingly widely recognized as a limitation of MR (Nitsch et al., 2006; Boef et al., 2015; Canan et al., 2017; Munafo et al., 2017; Swanson et al., 2017; Gkatzionis and Burgess, 2018; Munafo and Smith, 2018; Vansteelandt et al., 2018; Hughes et al., 2019; Swanson, 2019), which may violate the instrumental variable assumptions. Sources of potential selection bias in MR have been specifically identified as selecting an unrepresentative sample (Munafo et al., 2017; Munafo and Smith, 2018; Hughes et al., 2019), attrition from an initially representative sample, such as a birth cohort (Munafo et al., 2017), and selecting a sample strongly on surviving the exposure (Gkatzionis and Burgess, 2018) or genotype of interest (Vansteelandt et al., 2018; Smit et al., 2019). What has not explicitly been considered is selecting the underlying sample(s) on surviving the genotype of interest in the presence of competing risk of the outcome. MR studies are particularly vulnerable to sample selection on survival because of the time lag between genetic randomization (at conception) and typical recruitment into genetic studies of major diseases in middle to old age. MR studies also often concern major causes of death thought to share considerable etiology. For example, lipids, blood pressure, diabetes, lifestyle (such as smoking, diet, physical activity, and sleep), and socioeconomic position cause both ischemic heart disease (IHD) and ischemic stroke, with death from IHD typically occurring at younger ages than death from stroke (Kesteloot and Decramer, 2008; Menotti et al., 2019). As a result, a study of the association of lipid modifiers with stroke among the living will automatically select on surviving high lipids and on surviving competing risk of prior death from IHD due to shared etiology between IHD and stroke. Some people dying from genetically high lipids and others dying from IHD before recruitment into a stroke study will leave a shortage of people available to recruit with genetically high lipids and susceptibility to stroke, thereby obscuring any effect of lipids or lipid modifiers on stroke. Correspondingly, MR studies suggest less effect of lipids and lipid modifiers on stroke than IHD (Hopewell et al., 2018; Valdes-Marquez et al., 2019), although RCTs suggest similar effects (Mills et al., 2011; Chou et al., 2016; Schmidt et al., 2017). Similarly, MR studies do not consistently show detrimental effects of body mass index (BMI) on stroke (Marini et al., 2020). In this study, we explain how potential violations of the instrumental variable assumptions due to inadvertently recruiting survivors of the genetically predicted exposure and competing risk of the outcome may bias MR estimates. We explain how this bias might be corrected using multivariable MR and provide a simple means of estimating how large the bias is likely to be.

## Materials and Methods

### Potential Biasing Pathways Due to Recruiting on Selective Survival

**FIGURE 1:**
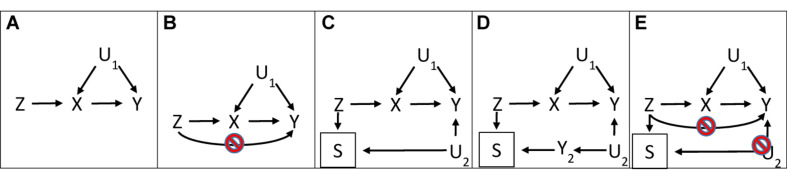
Directed acyclic graphs with instrument (Z), outcome (Y), exposure (X), confounders (U_1_), and survival (S), where a box indicates selection, for **(A)** a valid Mendelian randomization study and **(B)** a Mendelian randomization study with an invalid instrument through violation of the exclusion-restriction assumption via pleiotropy, **(C)** a Mendelian randomization study with an invalid instrument through violation of the exclusion-restriction assumption via survival on instrument and shared etiology with the outcome (U_2_), **(D)** a Mendelian randomization study with an invalid instrument through violation of the exclusion restriction assumption via survival (S), competing risk of another disease (Y_2_) and shared causes (U_2_) with (Y_2_) and the outcome (Y), and **(E)** a Mendelian randomization illustrating both conditions which have to be met to satisfy the exclusion restriction assumption.

**FIGURE 2:**
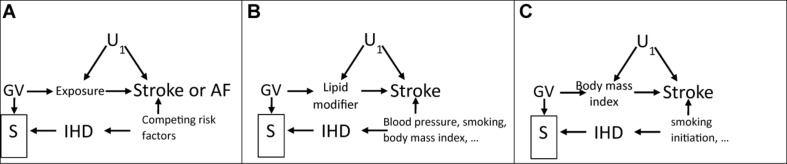
Directed acyclic graphs showing how selection bias could occur because of selection on survival (S), indicated by a box, on the instrument (GV) and on competing risk of ischemic heart disease (IHD) which shares causes with the outcome of interest, i.e., stroke, with U_1_ as confounders of exposure and outcome, when assessing **(A)** effects of an exposure on stroke or AF, **(B)** effects of lipid modifiers on stroke, and **(C)** effects of body mass index on stroke.

Figure 1A shows the directed acyclic graph for MR illustrating the instrumental variable assumptions typically referred to as relevance, independence, and exclusion restriction. Relevance is explicitly indicated by the arrow from instrument to exposure. Independence is implicitly indicated by the lack of an arrow from confounders of exposure on outcome (or of instrument on outcome) to instrument. Exclusion restriction is implicitly indicated by the lack of arrows linking instrument to outcome, sometimes illustrated as no arrow from instrument to outcome indicating no pleiotropy (Bowden et al., 2015, 2016; Hartwig et al., 2017; Verbanck et al., 2018) (Figure 1B). Figure 1C shows selection on survival of both instrument and common causes of the outcome (U_2_) (Hughes et al., 2019; Swanson, 2019), which also violates the exclusion restriction assumption, particularly when stated as “*every unblocked path connecting instrument and outcome must contain an arrow pointing into the exposure*” (Pearl, 2009). Figure 1D explicitly shows survival on instrument, and another disease (Y_2_) sharing etiology (U_2_) with the outcome (Y). Figure 1E shows the exclusion restriction assumption with both no pleiotropy and no selection bias from competing risk (U_2_) made explicit. Notably, Figures 1C–E are very similar in structure to a well-known example of selection bias, which occurs when conditioning on an intermediate (or covariable adjustment) reverses the direction of effect: the “birth weight” paradox (Hernandez-Diaz et al., 2006). In the birth weight paradox adjusting the association of maternal smoking with infant death for birth weight makes maternal smoking look protective; further adjusting for all common causes of birth weight and infant death, thought to be birth defects, should remove this bias (Hernandez-Diaz et al., 2006) by blocking the path from maternal smoking to infant death via birth weight and birth defects. Similarly, bias due to inadvertently selecting the underlying sample in an MR study on surviving the genetically instrumented exposure and surviving competing risk of the outcome should be ameliorated by adjusting for major causes of survival and the outcome (Figure 2). The recent development of multivariable MR (Sanderson et al., 2019) provides the means to do so. Specifically, as indicated in Figures 1C,D, where univariable MR may be biased, using multivariable MR adjusting for the main determinants of survival and outcome may reduce bias by at least partially blocking any backdoor paths from instrument to outcome.

In addition, to provide triangulation, the level of selection bias due to surviving to recruitment on genetically instrumented exposure in the presence of competing risk of the outcome can also be thought of as depending on the proportion of the exposed who are not available for recruitment because of prior death due to the genetically predicted exposure and the proportion of those who could have experienced the outcome who are not available for recruitment because of prior death from a competing risk. Assuming these proportions are independent and their corresponding probabilities do not sum to more than 1, then for an observed odds ratio (OR) greater than 1, the true OR for genetically predicted exposure on disease can be estimated as the observed OR multiplied by the ratio of the probability of surviving the exposure and the competing risk to the probability of surviving the exposure or the competing risk, as shown in Appendix Table 1.

### Examples of Selection Bias and Amelioration

We investigated effects of lipid modifiers and BMI on ischemic stroke as possible exemplars, because previous MR studies of these exposures on stroke have not always given the expected results (Hopewell et al., 2018; Marini et al., 2020). Statins and PCSK9 inhibitors are very well-established interventions for cardiovascular disease, which reduce low-density lipoprotein (LDL)-cholesterol, IHD (Mills et al., 2011; Chou et al., 2016; Schmidt et al., 2017), stroke (Mills et al., 2011; Chou et al., 2016; Schmidt et al., 2017), and atrial fibrillation (AF) (Peng et al., 2018). BMI is also known to be harmful. IHD, stroke, and AF also share major causes independent of lipid modifiers, such as blood pressure (Emdin et al., 2015; Ettehad et al., 2016), smoking, lifestyle, and socioeconomic position. Death from IHD typically occurs at earlier ages than death from stroke in Western populations (Kesteloot and Decramer, 2008; Menotti et al., 2019). AF may also be a consequence of IHD. Figure 2A suggests bias would be expected for harmful exposures on stroke or AF in any sample of survivors, such as middle-aged or older adults. Adjusting for major factors causing survival to recruitment into the underlying studies of stroke or AF, as shown for lipid modifiers on stroke (Figure 2B) or BMI on stroke (Figure 2C), should reduce the bias. As such, univariable MR, even with well-defined genetic instruments free from genetic pleiotropy, might generate biased estimates due to selection bias violating the exclusion-restriction assumption, but appropriate use of multivariable MR might ameliorate the problem.

We used well-established independent genetic variants to mimic effects of statins (rs12916) and proprotein convertase subtilisin/kexin type 9 (PCSK9) inhibitors (rs11206510, rs2149041, and rs7552841) (Ference et al., 2019), and for BMI (96 variants) (Locke et al., 2015). Using two-sample univariable MR, we applied these variants to major GWAS, in people largely of European descent, of IHD (CARDIoGRAMplusC4D 1000 Genomes) (Nikpay et al., 2015), stroke (MEGASTROKE) (Malik et al., 2018), and AF (Nielsen et al., 2018). We also used the UK Biobank summary statistics for IHD and stroke (Zhou et al., 2018), but not for AF because the AF GWAS includes the UK Biobank data (Nielsen et al., 2018). We obtained univariable MR estimates by meta-analyzing the Wald estimates (genetic variant on outcome divided by genetic variant on exposure) using inverse variance weighting, with multiplicative random effects, after aligning variant estimates on the same-effect allele in each study.

We used multivariable two-sample MR to obtain MR estimates for the lipid modifiers on stroke and AF adjusted for major causes of survival (smoking initiation, blood pressure, and BMI) (Forouzanfar et al., 2015; Sakaue et al., 2020) and stroke, and to obtain an MR estimate for BMI on stroke adjusted for smoking initiation. We used published independent genetic instruments for smoking initiation (327 variants) (Larsson et al., 2020), systolic blood pressure (SBP) and diastolic blood pressure (DBP) [all replicated variants (SBP 215, DBP 219)] (Evangelou et al., 2018), and BMI (96 variants) (Locke et al., 2015). Genetic associations, for all the instruments selected, with LDL-cholesterol, ever smoking, SBP, DBP, and BMI, were obtained from the UK Biobank summary statistics^1^ adjusted for age, sex, age^2^, sex^∗^age, and sex^∗^age^2^ and the first 20 principal components. We used the MR-Base clump_data R package with *r*^2^<0.05 to obtain independent genetic variants across exposures and the MendelianRandomization package to obtain IVW multivariable estimates. Here, we used summary statistics, meaning we assumed linear and homogenous effects for all exposures. We reported the multivariable conditional F-statistic as a measure of instrument strength and the multivariable Q-statistic as a measure of instrument pleiotropy (Sanderson et al., 2019), obtained using the MVMR package (Sanderson et al., 2019). Calculation of the conditional F-statistic and the multivariable Q-statistic requires the covariance between the effects of genetic variants on each exposure or use of non-overlapping samples for the exposure GWAS (Sanderson et al., 2019). Use of summary statistics for the exposures makes it difficult to obtain their covariance, so we largely selected genetic instruments for exposures from non-overlapping samples; however, some overlap exists, for example, the GWAS used to obtain genetic instruments for smoking initiation and blood pressure both included the UK Biobank (as 33 and ∼40% of the sample, respectively) (Forouzanfar et al., 2015; Locke et al., 2015; Evangelou et al., 2018; Larsson et al., 2020; Sakaue et al., 2020). As such, the conditional F-statistic gives a lower bound for strength of the instruments and the modified Q-statistic gives an upper bound on bias from pleiotropy (Sanderson et al., 2019). Notably, in this context, a significant multivariable Q statistic may indicate genetic pleiotropy or violation of the exclusion restriction assumption by selection bias, because both might inflate the multivariable Cochran Q. If the same instruments give very different multivariable Cochran’s Q for the same outcomes in different studies or for related outcomes in the same study, it would suggest that estimates with higher Cochran’s Q are more likely open to selection bias than genetic pleiotropy. We also reported the multivariable MR-Egger intercept which may indicate genetic pleiotropy (Rees et al., 2017).

This study only used publicly available genetic summary statistics, collected with consent, and so does not require ethical approval.

## Results

As expected, the cases recruited into the underlying GWAS (Nikpay et al., 2015; Malik et al., 2018; Nielsen et al., 2018) seemed to be youngest for IHD and oldest for AF with stroke somewhere in between (Supplementary Table 1). In univariable MR, genetically mimicking statins or PCSK9 inhibitors reduced IHD, while genetically instrumented BMI increased IHD (Table 1). Estimates were similar using CARDIoGRAMplusC4D 1000 Genomes and the UK Biobank. IHD is not expected to be majorly open to competing risk, so it was not considered further. In univariable MR, genetically mimicking statins or PCSK9 inhibitors was not associated with a lower risk of stroke or AF; some estimates for statins were in the direction opposite to expected (Table 1). In univariable MR, genetically instrumented BMI did not consistently increase stroke but did increase AF (Table 1). Univariable MR estimates for the major causes of survival considered are shown in Supplementary Table 2.

**TABLE 1:** Effect of genetically mimicking statins and PCSK9 inhibitors use (Ference et al., 2019) (in effect size of LDL-cholesterol) and BMI (Locke et al., 2015) on IHD using the CARDIoGRAMplusC4D 1000 Genomes based GWAS (Nikpay et al., 2015) and the UK Biobank on all ischemic stroke using MEGASTROKE (Malik et al., 2018) and the UK Biobank and on AF using a study by Nielsen et al. (2018) from univariable Mendelian randomization and from multivariable Mendelian randomization, with genetically mimicked statins and PCSK9 inhibitors adjusted for systolic blood pressure (Evangelou et al., 2018), diastolic blood pressure (Evangelou et al., 2018), smoking initiation (Larsson et al., 2020) and BMI (Locke et al., 2015), and BMI adjusted for smoking initiation.

			Univariable		Multivariable				
Disease	Source of genetic associations with disease	Exposure	OR	95% CI	OR	95% CI	MR-Egger Intercept	Conditional F	Q *p*-value
Ischemic heart disease	CARDIoGRAMplusC4D 1000 Genomes	Statin	0.56	0.41–0.75					
		PCSK9 inhibitor	0.32	0.22–0.46					
		BMI	1.57	1.36–1.81					
	UK Biobank (SAIGE)	Statin	0.69	0.52–0.93					
		PCSK9 inhibitor	0.47	0.34–0.65					
		BMI	1.38	1.18–1.61					
All ischemic stroke	MEGASTROKE	Statin	1.17	0.84–1.65	1.05	0.91–1.21	0.56	5.8	1.4e–10
		PCSK9 inhibitor	0.94	0.65–1.37	1.02	0.88–1.18	0.47	5.7	1.3e–10
		BMI	1.18	1.04–1.34	1.16	1.05–1.28	0.12	18.0	0.0001
	UK Biobank (SAIGE)	Statin	1.33	0.80–2.20	0.79	0.65–0.97	0.04	5.8	0.09
		PCSK9 inhibitor	0.96	0.55–1.69	0.76	0.62–0.92	0.02	5.7	0.11
		BMI	1.13	0.93–1.36	1.27	1.10–1.47	<0.001	18.0	0.02
Atrial fibrillation	Nielsen et al. (including UK Biobank)	Statin	1.22	0.97–1.54	1.16	1.03–1.32	0.02	5.9	1.3e–80
		PCSK9 inhibitor	0.79	0.62–1.01	1.12	0.99–1.27	0.01	5.7	1.3e–81
		BMI	1.46	1.34–1.59	1.44	1.35–1.56	0.70	17.9	4.6e–18

In multivariable MR, the conditional F-statistics for each exposure were similar in each analysis, suggesting similar instrument strength (Table 1). The Q-statistics were not significant for lipid modifiers on UK Biobank stroke (Table 1). The multivariable MR estimates in the UK Biobank, in contrast to the corresponding univariable MR estimates, showed that genetically instrumented lipid modifiers protected against stroke and that genetically instrumented BMI caused stroke (Table 1). The multivariable MR-Egger intercepts were significant, with largely similar MR-Egger estimates for statins [OR 0.70, 05% confidence interval (CI) 0.56–0.88] and PCSK9 inhibitors (OR 0.66, 95% CI 0.53–0.83) but not BMI (OR 1.00, 95% CI 0.83–1.20). The Q-statistics were highly significant for lipid modifiers and BMI on MEGASTROKE stroke and AF (Table 1), indicating that these estimates were likely still biased by pleiotropy probably from selection bias given the same instruments gave estimates apparently unbiased by genetic pleiotropy for stroke in the UK Biobank. Correspondingly, the multivariable MR estimates were similar to the univariable estimates, and for lipid modifiers differed from those expected from RCTs (Table 1). The multivariable MR-Egger intercepts were not significant for MEGASTROKE estimates or for BMI on AF but were significant for statins and PCSK9 inhibitors on AF. The corresponding multivariable MR-Egger estimates gave directionally similar estimates to the inverse variance weighted estimates for genetically mimicked statins (OR 1.06, 95% CI 0.92–1.23) and PCSK9 inhibitors (OR 1.01, 95% CI 0.87–1.17).

To provide triangulation, we estimated whether the level of selection bias for statins on stroke, from surviving genetically instrument statins and IHD, was consistent with the univariable estimate, using the formula given in Appendix Table 1. The OR for the protective allele of the statin single-nucleotide polymorphism (rs12916) on IHD used to obtain the Wald estimate was 0.96. Assuming statins have the same effect on IHD and stroke, it would only take 10% with that harmful allele and 25% of potential stroke cases to have died from IHD or other competing risks before recruitment into a stroke study for the observed OR to be exactly 1.0, which would give a null MR estimate. If instead 40% of potential stroke cases had died from competing risk before recruitment, then the OR would reverse to 1.04 and give an MR estimate similar to the univariable estimate from MEGASTROKE.

## Discussion

Here, we have shown theoretically, empirically, and analytically that univariable MR studies can be open to quite severe selection bias likely arising from selective survival on genetically instrumented exposure when other causes of survival and outcome exist, i.e., competing risk before recruitment. We have also explained the relevance of this situation to the assumptions of MR, as a violation of the exclusion restriction assumption, how to mitigate this bias using multivariable MR, how to assess the success of this mitigation (using the multivariable Q statistic), and how to make an assessment of the possible level of bias using an approximation based on contextual knowledge (Appendix Table 1). Notably, genetic studies are particularly vulnerable to bias because most genetic estimates are of small magnitude; the closer the true estimate is to the null, the easier it is for a reversal to occur (Appendix Figure 1).

Our study differs from many other studies suggesting that MR is open to selection bias by specifically identifying when such bias can occur in the context of a typical MR study using existing GWAS, and by showing how any such bias may be addressed along with a means of checking whether the bias has been successfully addressed. For participants selected on surviving the genetically instrumented exposure and competing risk of the outcome, our study is similar to other studies about bias in MR in showing that bias can occur from using GWAS summary statistics with “covariable adjustment” (Hartwig et al., 2020). We add by explaining that selecting from the living is common in MR studies and may engender covariable adjustment on survival. Rather than suggesting that such situations should be avoided (Hartwig et al., 2020), precluding MR studies of a harmful exposure on a late-onset disease subject to competing risk, we show how such situations can be addressed. Specifically, external knowledge can be used to identify potential common causes of survival and outcome, followed by multivariable MR to adjust for them and thereby possibly obtain a less biased estimate, bearing in mind the Q statistic. We also show that when, in this situation, it is not possible to adjust comprehensively for factors causing survival and the outcome, the level of potential bias can be estimated (Appendix Table 1). Alternatively, restricting MR studies to younger people will usually reduce bias because death prior to recruitment is less common in younger people. However, these studies may need to consider competing risk after recruitment. Our study also implies that care should be taken in interpreting phenome-wide association studies identifying the effect of a specific genetically instrumented exposure across the phenome, because the effects of harmful exposures observed will vary depending of the level of competing risk of the outcome.

Despite the strengths of our study in explicating and providing means of addressing a relatively common bias in univariable MR, limitations exist. First, use of multivariable MR to address bias arising from sample selection on survival requires knowledge of the underlying causal structure and suitable genetic instruments for all sources of bias. In all observational studies, knowledge of the underlying causal structure is needed to identify potential sources of confounding and selection bias. For example, here our results could also be due to removing the harmful effects of statins and PCSK9 inhibitors via body composition by adjusting for BMI, although these effects are still under investigation (Nelson et al., 2019). Alternative methods to recover from selection bias due to surviving the genetically instrumented exposure and competing risk of the outcome that do not require knowledge of the underlying causal structure or additional data would be easier to use. Second, our study did not conduct simulations of the level of bias. Simulations including research questions with the same underlying directed acyclic graph s as investigated here have been done (Hartwig et al., 2020), and simulation of a similar situation is available (Glymour and Vittinghoff, 2014). The key issue in making use of these simulations is appreciating when these biasing situations might arise and how serious the issues can be in practice, which is the gap addressed by this study. As such, we address appreciating which real-life situations will result in the simulated bias, and what to do to ameliorate it. Third, we provide a means of addressing any such selection bias using multivariable MR (adjusting for common causes of survival and outcome) as well as a means of assessing the likely validity of the revised estimate (non-significant multivariable Q-statistic). However, application and interpretation may not always be straightforward. As with any bias correction by adjustment, it may not be feasible to recover the correct estimate, due to lack of contextual knowledge, a highly interrelated causal structure, such as the genetic instruments causing common causes of survival and outcome, or a lack of relevant information. Fourth, we also provide an approximation to estimate the likely effects of such bias (Appendix Table 1). However, given that the role of selection bias due to death before recruitment from the genetically predicted exposure or from a competing risk of the outcome has rarely been explicitly considered previously, the information needed to identify the sources of bias and estimate the likely level of bias is not easily available. More research concerning the effects of genetic exposures on longevity and the sequence of death from different diseases in different populations would be helpful, as well as easily accessible information about the age and sex structure of participants in genetic studies by case status. Fifth, we do not provide an exhaustive list of examples of when this bias has occurred, because few MR studies have been validated against RCTs. For example, Alzheimer’s disease usually occurs in old age and appears to share causes with determinants of longevity (Deelen et al., 2019), so MR studies of harmful exposures on Alzheimer’s disease could be open to selection bias but the true causes of Alzheimer’s disease are unknown making any determination of whether the MR studies are biased or not difficult. Finally, the issue of obtaining valid estimates in the presence of selective survival on exposure and competing risk of the outcome is similar to the issue of obtaining valid genetic estimates in other studies of survivors, i.e., patients. The current solution for obtaining valid estimates in genetic studies of patients relies on the assumption that the factors causing disease and disease progression differ (Dudbridge et al., 2019). Use of multivariable MR to adjust observational studies in patients suitably might bear consideration.

Specifically, as regards the example here, for the MR estimate for statins on stroke, we were able to recover a plausible estimate in the UK Biobank but not in MEGASTROKE. The UK Biobank participants are younger (∼57 years) than the MEGASTROKE participants (Supplementary Table 1), so the confounders of survival to recruitment and stroke used to adjust for survival could also be more biased by survival in MEGASTROKE making adjustment less effective in MEGASTROKE than in the UK Biobank, possibly as indicated by Supplementary Table 2. In addition, the Q-statistic represents both genetic pleiotropy and pleiotropy due to selection bias, so it is possible that the Q-statistic in MEGASTROKE is larger due to MEGASTROKE having more cases than UK Biobank rather than more severe selection bias, although the same instruments were used in both studies. The conditional F-statistics were quite low for lipid modifiers; however, they did not differ by outcome, so they are unlikely to fully explain the difficulty in fully recovering plausible estimates. The multivariable Q-statistics could also be somewhat larger because some samples used to obtain instruments for the exposures overlapped (Sanderson et al., 2019). However, given the very large Q-statistics for the multivariable estimates for stroke using MEGASTROKE and for AF (Table 1), this overlap is unlikely to affect the interpretation. Finally, the multivariable MR-Egger intercepts were not always significant even when the estimates did not look plausible, perhaps because MR-Egger detects exposure specific directional pleiotropy. In contrast, the multivariable Q-statistic assesses heterogeneity across several exposures which if different due to differing selection bias by exposure could contribute to a larger multivariable Cochran’s Q as well as biased estimates.

## Conclusion

Here, we have shown theoretically, empirically, and analytically that univariable MR studies can be open to quite severe selection bias arising from selecting on survival of genetically instrumented exposure when other causes of survival and outcome exist, i.e., competing risk before recruitment. Bias from such selection bias is likely to be least for MR studies of harmless exposures recruited shortly after genetic randomization with no competing risk, i.e., studies using birth cohorts with minimal attrition. Conversely, such bias is likely to be most evident for MR studies recruited at older ages examining the effect of a harmful exposure on an outcome subject to competing risk from shared etiology with other common conditions that occur earlier in life. Use of multivariable MR to adjust for major causes of survival and outcome may ameliorate this bias, while simple sensitivity analysis based on information about the exposure and the natural history of disease may help quantify the magnitude of the bias. Infallible, methods of obtaining valid MR estimates, when the exclusion restriction is invalidated by selection bias stemming from competing risk, that do not require external knowledge, would be helpful.

## Data Availability Statement

The original contributions presented in the study are included in the article/Supplementary Material. Further inquiries can be directed to the corresponding author/s. This study only uses publicly available R packages to conduct the analysis. The code used to arrange the data for analysis is available on request.

## Ethics Statement

This study only used publicly available genetic summary statistics, collected with consent, and so does not require ethical approval.

## Author Contributions

CS originated the study concept. PL, SAY, and JH explicated the concepts. JZ and ZY contributed substantially to the analysis, and implementation of the concepts. PL and CS wrote the first draft. SAY and JH contributed to the interpretation and presentation. All authors contributed to drafting and revising the article for intellectual content and approved the final version. All authors are accountable for all aspects of the work.

## Conflict of Interest

The authors declare that the research was conducted in the absence of any commercial or financial relationships that could be construed as a potential conflict of interest.

## Figures and Tables

**FIGURE A1:**
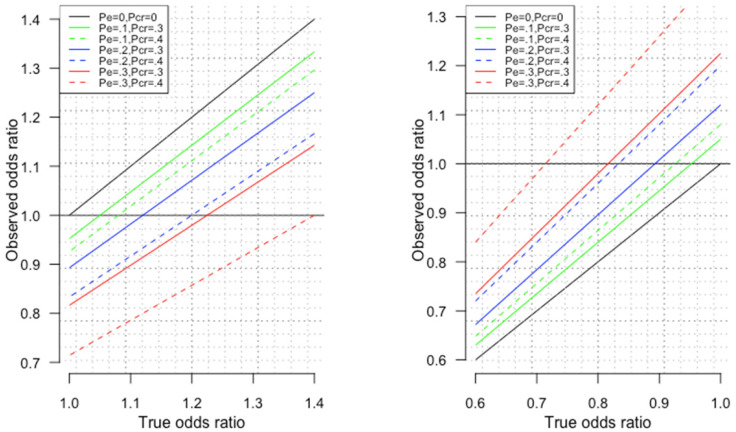
Observed odds ratio against the True odds ratio in the presence of different proportions of death before recruitment due to the exposure (P_*E*_) and different proportions of death before recruitment due to competing risk of the outcome (P_*CR*_) for true odds ratios large than 1 (left hand side) and smaller than 1 (right hand side, obtained by taking the inverse of the odds ratio).

## Footnotes

1 http://www.nealelab.is/uk-biobank

## Appendix

A possible solution for recovering the causal effect in the presence of selection bias due to selecting on surviving the exposure and competing risk of the exposure in a case-control study.

The fundamental issue of the selection bias in a case–control study is unknown information for the “missing” (or unselected) participants. Appendix Table 1 shows the possible mechanism generating a biased causal effect due to selection on surviving the exposure (E) and surviving competing risk (CR) of the outcome (D) in a case–control study.

**TABLE A1:** Possible mechanism for biased causal effects in a case-control study due to selection bias from surviving the exposure and competing risk of the outcome.

		S = 1		S = 0	
		D = 1	D = 0	D = 1	D = 0
E = 1	CR = 1	a′	b′	P_*E*_	P_*E*_ + P_*CR*_
	CR = 0				
E = 0	CR = 1	c′	d′	0	P_*CR*_
	CR = 0				
Observation		OR^*obs*^			
Target		OR^*true*^			

*S indexes selection status of participants, i.e., S = 1 indicates those selected and S = 0 indicates those unselected. D indexes outcome status, i.e., D = 1 indicates disease and D = 0 indicates no disease. E indexes exposure status, i.e., E = 1 indicates the exposed, and E = 0 indicates unexposed. CR indicates competing risk (CR) of the outcome D; i.e., CR = 1 then D = 0, and if D = 1 and CR = 0. a′, b′, c′, and d′ are observed data about the selected participants.*

Based on the observed data a′, b′, c′, and d′, the observed causal effect of E on D using an OR (^ORobs^) is,

$${\text{OR}}^{\text{obs}}=\frac{a^{\prime }/b^{\prime }}{c^{\prime }/d^{\prime }}=\frac{a^{\prime }\,d^{\prime }}{b^{\prime }\,c^{\prime }}$$

To obtain the true causal effect, we have to recover the data for the whole population, i.e., the birth cohorts who formed the population. Let P_*E*_ denote the proportion of participants unselected due to E, and let P_*CR*_ denote the proportion of participants unselected due to CR. Suppose P_*E*_ and P_*CR*_ are additive, and 0 < P_*E*_ + P_*CR*_ < 1. We can construct the pattern of the unselected participants, as shown in Appendix Table 1. As such, the causal effect of E on D for the whole population can be estimated as follows,

$${\text{OR}}^{\text{true}}=\frac{\text{ad}}{\text{bc}}=\frac{\frac{{\text{a}}^{{}^{\prime }}/\left(1-P_{E}\right)}{{\text{b}}^{{}^{\prime }}/\left(1-P_{E}-P_{C\,R}\right)}}{\frac{{\text{c}}^{{}^{\prime }}}{{\text{d}}^{{}^{\prime }}/\left(1-P_{C\,R}\right)}}={\text{OR}}^{\text{obs}}\times \frac{\left(1-P_{E}\right)\,\left(1-P_{C\,R}\right)}{\left(1-P_{E}-P_{C\,R}\right)}$$

This relationship will be invalid if we replace the OR with a risk ratio.

Notably, the level of bias depends on the magnitude of the OR. A small OR, of the order of 1.05, as is typical in a genetic study, is much more vulnerable to a reversal of effect from selection bias due to selecting on surviving the exposure and surviving competing risk of the outcome than a larger OR, of the order of 1.50, as is typical in traditional observational studies. To clarify Appendix Figure 1 shows the observed OR plotted against the true OR for different combinations of selection on survival (P_*E*_) and selection on competing risk of surviving the outcome (P_*CR*_).
