# Supplementary material for: Use of Multivariable Mendelian Randomization to Address Biases Due to Competing Risk Before Recruitment
Source: Front Genet. 2021 Jan 15;11:610852. doi: 10.3389/fgene.2020.610852 (PMC7845663; doi:10.3389/fgene.2020.610852)
Supplement: Supplementary file 1 [file Data_Sheet_1.pdf]

Supplementary Material

Supplementary table 1: Study details for the GWAS of IHD, stroke and AF

Study	Phenotype (Phewas code)	Cases	Non-cases	Mean age of cases	Phenotype definition	Adjusted for (non-genetic)
Cardiogram 1000 genomes GWAS [43]	Ischemic heart disease	60,801	123,504	n/a, possibly ~58 years	"Case status was defined by an inclusive CAD diagnosis (e.g. myocardial infarction (MI), acute coronary syndrome, chronic stable angina, or coronary stenosis >50%)"	Study-specific covariates (not age or sex)
UK biobank SAIGE [46]	CAD (411)<br>Stroke (433)	31,355<br>8,742	377,103<br>399,017	n/a	Phewas code based on self-report, hospital episodes and death	Sex, birth year, and principal components 1 to 4
MEGASTROKE [44]	All ischemic stroke	60,341	454,450	~69 years	Several different definitions used	Minimum of age and sex
AF [45]	Atrial fibrillation	60,620	970,216	~74 years	Usually based on ICD-9 427.3 and ICD-10 I48	Minimum of age (birth year) and sex

Outcome	Source	Exposure	Odds ratio	95% Confidence Interval
Ischemic heart disease	CARDIoGRAM plusC4D 1000 Genomes	SBP	2.01	1.69 to 2.39
		DBP	1.62	1.38 to 1.92
		Smoking initiation	2.19	1.65 to 2.92
		BMI	1.57	1.36 to 1.81
	UK Biobank	SBP	2.17	1.85 to 2.56
		DBP	1.57	1.33 to 1.85
		Smoking initiation	4.13	3.08 to 5.55
		BMI	1.38	1.18 to 1.61
All ischemic stroke	MEGASTROKE	SBP	1.84	1.54 to 2.19
		DBP	1.42	1.21 to 1.67
		Smoking initiation	2.10	1.60 to 2.76
		BMI	1.18	1.04 to 1.34
Stroke	UK Biobank	SBP	2.28	1.83 to 2.84
		DBP	1.55	1.25 to 1.93
		Smoking initiation	3.93	2.63 to 5.87
		BMI	1.12	0.93 to 1.36
AF	Nielsen	SBP	1.55	1.35 to 1.77
		DBP	1.16	0.97 to 1.39
		Smoking initiation	1.56	1.24 to 1.96
		BMI	1.46	1.34 to 1.59

Supplementary Table 2: Univariable MR inverse variance weighted estimates for systolic blood pressure (SBP) [50], diastolic blood pressure (DBP) [50], smoking initiation [47] and BMI [42] on IHD using the CARDIoGRAMplusC4D 1000 Genomes based GWAS [43] and the UK Biobank, on all ischemic stroke using MEGASTROKE [44] and the UK Biobank and on AF using a study by Nielsen et al [45]
